# Materia Medica, Pharmacology and Therapeutics

**Published:** 1891-09

**Authors:** 


MATERIA MEDICA, PHARMACOLOGY AND THERAPEUTICS. Shoemaker. Vol. II. F. A. Davis. Philadelphia.

The first, or introductory volume contained but 350 pages; the last, 1,000: It is so written that it may be used independent of the first volume, which, in a previous review, however, we criticised so favorably. Next to Potter's Materia Medica and Therapeutics, we regard it as the best work that has appeared on the subject in a decade. While not so scientific as Brunton's work for the average practitioner and the medical student, it, in our opinion, is much superior, and we predict that the sale of the excellent book will verify our estimate of its value and appreciation by the general profession.

A painstaking care marks nearly every article, and it is of the latest; as an example of which, six and a-half pages are devoted to tuberculinum—Koch's lymph. We quote the last sentence on this important subject: "In several other cases the apparent result of the injection is good, for the present, upon the lesions; but time alone can decide the ultimate effect in tuberculosis, lupus, and other diseases."

We hope that when a second edition is issued—and we predict that one will be demanded soon—that the author will give the prescriptions, of which there are a multitude, rather in plain English than couched in the "dog Latin" which mars at least fifty per cent. of them in the volume before us.

